# Key point generation as an instrument for generating core statements of a political debate on Twitter

**DOI:** 10.3389/frai.2024.1200949

**Published:** 2024-03-20

**Authors:** Philip Ehnert, Julian Schröter

**Affiliations:** ^1^iits-consulting/ImpressSol GmbH, Department of Artificial Intelligence, Au in der Hallertau, Germany; ^2^FOM—Hochschule für Oekonomie und Management GmbH, Department of Business Informatics, Bonn, Germany

**Keywords:** key point generation, topic modeling, abstractive summarization, hyperparameter tuning, semantic textual similarity

## Abstract

Identifying key statements in large volumes of short, user-generated texts is essential for decision-makers to quickly grasp their key content. To address this need, this research introduces a novel abstractive key point generation (KPG) approach applicable to unlabeled text corpora, using an unsupervised approach, a feature not yet seen in existing abstractive KPG methods. The proposed method uniquely combines topic modeling for unsupervised data space segmentation with abstractive summarization techniques to efficiently generate semantically representative key points from text collections. This is further enhanced by hyperparameter tuning to optimize both the topic modeling and abstractive summarization processes. The hyperparameter tuning of the topic modeling aims at making the cluster assignment more deterministic as the probabilistic nature of the process would otherwise lead to high variability in the output. The abstractive summarization process is optimized using a Davies-Bouldin Index specifically adapted to this use case, so that the generated key points more accurately reflect the characteristic properties of this cluster. In addition, our research recommends an automated evaluation that provides a quantitative complement to the traditional qualitative analysis of KPG. This method regards KPG as a specialized form of Multidocument summarization (MDS) and employs both word-based and word-embedding-based metrics for evaluation. These criteria allow for a comprehensive and nuanced analysis of the KPG output. Demonstrated through application to a political debate on Twitter, the versatility of this approach extends to various domains, such as product review analysis and survey evaluation. This research not only paves the way for innovative development in abstractive KPG methods but also sets a benchmark for their evaluation.

## 1 Introduction

The rapidly growing amount of user-generated content on platforms such as Twitter has created new opportunities and challenges in the field of political discourse analysis. Recognizing the potential of such interactions and the vast availability of textual data, previous research has underlined the importance of these engagements for data-driven political and economic decision-making (Zeng et al., 2010; Stieglitz and Dang-Xuan, 2013). However, the growing amount of data and interactions also brings challenges, in particular the problem of information overload. In order to effectively address this problem and unlock the full potential of large amounts of text data, the use of big data analytics methods is increasingly recommended (He et al., 2017). This research seeks to tackle this problem by introducing an unsupervised abstractive KPG (Bar-Haim et al., 2020a) approach tailored to user-generated content on Twitter. The primary objective of key point analysis (KPA) is to identify and categorize core statements from large document collections of short, user-generated texts, thereby generating a comprehensive list of key points that reflect the dominant topics within the text collection. The result is a list of all identified key points, including the number of statements each key point represents to quantify its share of the debate.

**Figure 1:**
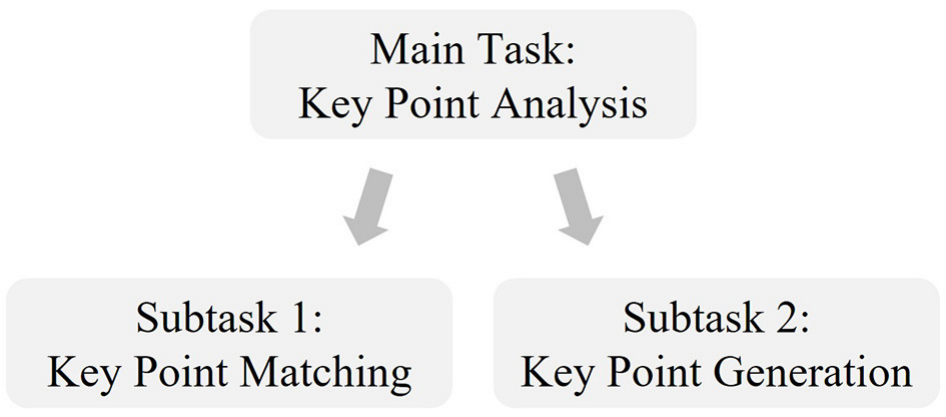
Overview of key point analysis and associated subtasks.

**Figure 2:**
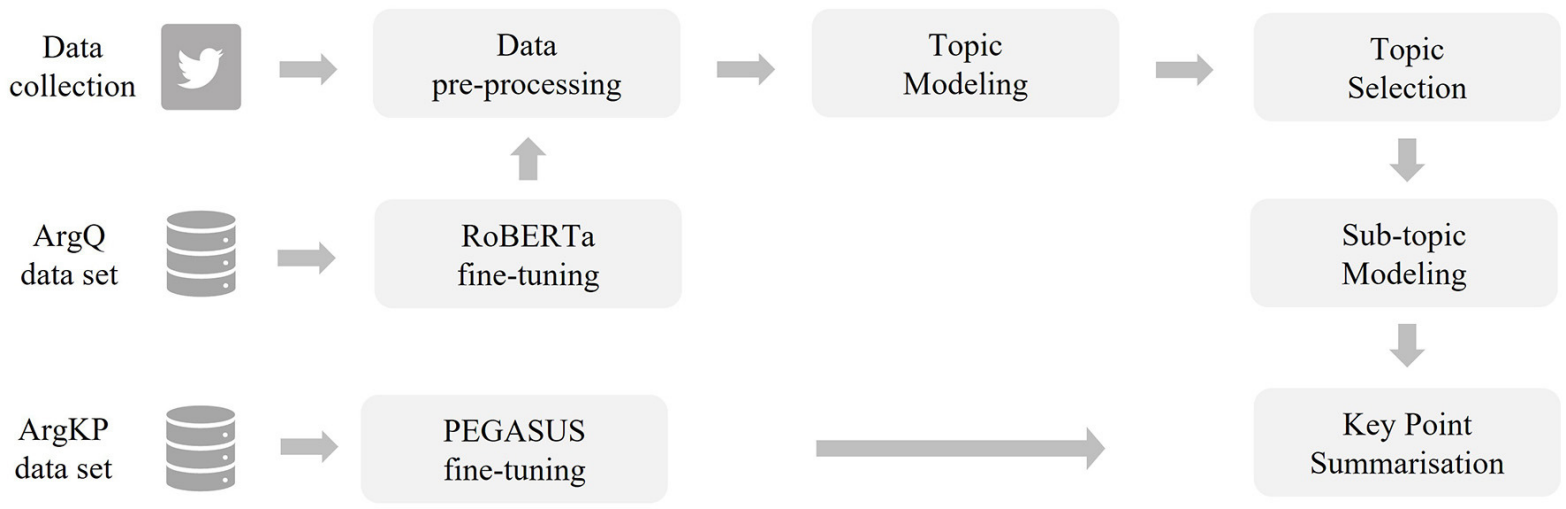
The training data sets used (see Section 2.1), the fine-tuning of language models (see Section 2.2), and the procedure of Twitter data preparation (see Section 2.3) provide the basis for the actual pipeline of key point generation (see Section 2.4). Starting with the selection of specific topics (see Section 2.4.1), through sub-topic modeling (see Section 2.4.2) to key point summarization (see Section 2.4.3).

The KPA task is divided into KPG and key point matching (KPM), as shown in Figure 1. KPG focusses on the identification and generation of key statements from a text corpus, while KPM involves the mapping of these statements back to the original corpus. Addressing the research question “How can an unsupervised abstractive key point generation method be developed and evaluated for application to unstructured, user-generated data in the context of political debates on Twitter?”, this study presents a novel approach to KPG and its evaluation. Unlike previous methods that rely on structured or specially annotated datasets, this research proposes an unsupervised KPG method that is tailored to the domain of political debate on Twitter. The first step is to prepare the Twitter data using a pre-processing pipeline to provide suitable data for a KPG. This is achieved by collecting tweets, applying pre-processing and topic modeling to identify high-level topics (see Figure 2). Our research innovatively integrates advanced topic modeling with hyperparameter tuning in the UMAP and HDBSCAN methods to achieve subtopic segmentation. Specifically, this tuning aims to make cluster assignment more deterministic, counteracting the inherent probabilistic variability of the process and ensuring more consistent output. In addition, we have developed a custom hyperparameter optimization for the abstractive summarization strategy. This strategy uses a modified Davies-Bouldin Index, specifically adapted for key point summarization, to improve the representativeness of the derived key points. Furthermore, we evaluated our KPG approach by considering it as a specialized form of MDS. In the future, this quantitative evaluation based on the word-based metric ROUGE (Lin, 2004) and the word-embedding-based metric BERTScore (Zhang T. et al., 2019) can be used to compare the performance of different approaches of KPG, even when applied to unlabeled data.

The subject of KPG is a relatively new area of research, but it has strong overlaps with established research areas such as MDS, opinion, and argument summarization (Friedman et al., 2021). Whereas, MDS deals with the concise summarization of a collection of heterogeneous text documents, argument summarization deals with identifying and subsequently summarizing argumentative content in highly opinionated text documents.

A distinction is also made between extractive and abstractive summarization methods. Extractive procedures of the MDS focus on using, for example, lexical (Landauer et al., 1998; Mihalcea and Tarau, 2004), graph-based (Erkan and Radev, 2004), or transformer-based (Liu, 2019; Zhang X. et al., 2019) methods to use representative sentences of the input documents as components of the generated summary. Abstractive methods, in contrast, use transformer-based generative language models, such as BART (Lewis et al., 2020) and PEGASUS (Zhang et al., 2020), to identify relevant content from various documents and summarize it in a concise, newly generated form.

Recent advancements in the optimization of these complex abstractive models have seen the application of meta heuristic techniques for hyperparameter tuning. Such approaches, exemplified by studies such as Bacanin et al. (2022), harness the power of meta heuristics to efficiently navigate the vast hyperparameter space. These methods demonstrate potential in significantly improving model performance, particularly in tasks requiring fine-tuned control over multiple parameters, which is often the case in abstractive summarization methods.

Previous research in the field of MDS has mainly focussed on the processing of longer text documents, summarizing newspaper articles (Fabbri et al., 2019), Wikipedia articles (Ghalandari et al., 2020), and scientific literature (Lu et al., 2020).

However, due to the lack of publicly available training data, summarization of short user-generated texts is underrepresented. Within the research area of MDS, the practicability of extractive and abstractive methods was evaluated in the context of summarizing posts on the Reddit platform (Sotudeh et al., 2021), product reviews (Angelidis et al., 2021; Oved and Levy, 2021), and Twitter streaming data (Dusart et al., 2023).

Following the definition of the research task of KPA (Bar-Haim et al., 2020a), first extractive approaches were developed to generate key points (Bar-Haim et al., 2020b, 2021), identifying high quality statements using a RoBERTa language model (Devlin et al., 2019; Liu et al., 2019) specifically adapted to this task. Finally, following the ArgMining Workshop 2021, a corresponding training and evaluation data set (Friedman et al., 2021) was published, explicitly designed for the development of new KPA methods. Based on this data set, further extractive approaches of KPG have been developed, which solve the problem via a graph-based method (Alshomary et al., 2021) or via the selection of representative key point candidates with the help of a combination of the evaluation metric MoverScore (Zhao et al., 2019) and the maximal marginal relevance (MMR) based on word embeddings of the statements (Shirafuji et al., 2021).

Although abstractive methods have the advantage of summarizing different semantic aspects of similar statements in a concise key point, they have been underrepresented in previous research. So far, only one approach has been developed to solve the KPG problem by using abstractive summarization with competitive performance (Friedman et al., 2021). For each statement in the text collection, key point candidates are generated using a language model (Zhang et al., 2020) that is specifically fine-tuned for this use case. Candidate key points are compared with expert annotated key points using the ROUGE evaluation metric, and only those with high scores are retained as final key points. Therefore, the approach described above is not applicable to unknown data.

## 2 Materials and methods

### 2.1 Data sets

#### 2.1.1 Argument quality data set

The generation of high-quality key points requires a basis of statements of high argumentative quality. To ensure the argumentative quality of tweets, the distinction between non-argumentative and argumentative statements has proved to be effective (Bosc et al., 2016; Schaefer and Stede, 2021). In the course of this research, characteristics of non-argumentative and argumentative tweets were learned using a language model based on the freely available data set *ArgQ* (Gretz et al., 2020). This data set containing a total of 30, 497 statements was generated with the help of annotators who were asked to write a pro and contra argument on a controversial discussion topic. The assessment of whether statements were argumentative or non-argumentative took the form of a binary annotation. For the quality of argumentation, two values are provided, the weighted average of all annotations and the MACE-P value (Habernal and Gurevych, 2016), which was also introduced to evaluate the argumentation quality of crowd annotations. The argument quality is given in the interval [0, 1] for both values.

#### 2.1.2 Key point summarization data set

To adapt language models to specific tasks, transfer learning using specialized training data sets has proven to be effective (Raffel et al., 2020). We used the *ArgKP*_2021 data set (Friedman et al., 2021), which was created specifically for the field of key point analysis. This data set is essentially a modified version of the *ArgQ* data set (Gretz et al., 2020). *ArgKP*_2021 consists of a total of 7, 238 statements on 31 topics and has been enriched with 276 key points through additional annotation by domain experts. Since each statement can be associated with several core statements, it consists of 27, 519 rows with information on the topic affiliation, the stance toward the topic, the actual statement text, and the associated key point.

### 2.2 Model fine-tuning

#### 2.2.1 Argument quality

In this research, the binary classification of non-argumentative and argumentative tweets is carried out via a fine-tuned RoBERTa-base model (Liu et al., 2019) using the Huggingface Transformers framework (Wolf et al., 2020). Following the approach of Bar-Haim et al. (2020a) to create a high-quality set of arguments, this research labeled statements from the *ArgQ* data set (Gretz et al., 2020) with a MACE-P argument quality value lower than 0.5 as non-argumentative. The binary labeled statements serve as training data for fine-tuning the model. The *f*1 value, which reflects the ratio of precision and recall achieved during the evaluation, was 0.785. The detailed procedure of the fine-tuning and the evaluation can be found in the Github repository associated with this publication.

#### 2.2.2 Key point summarization

This research uses an abstractive summarization technique to summarize similar statements. For this purpose, we use a PEGASUS language model since it can generate summaries of high quality with respect to the ROUGE score (Lin, 2004) achieved with only a few training samples for fine-tuning (Zhang et al., 2020). In addition to this property, we used a variant of the PEGASUS model pre-trained on the XSUM data set (Narayan et al., 2018). The XSUM data set consists of BBC articles and their highly condensed single-sentence summaries, making it ideal for generating key points. In our research, the fine-tuning is carried out using the *ArgKP*_2021 data set with statements from 25 of the 31 topics in the data set used for training. Participants of the ArgMining workshop used the concatenation of topic and statement as input and the corresponding key point as output to fine-tune the language model for the described abstractive approach (Friedman et al., 2021). In contrast, our research uses the concatenation of all statements explicitly assigned to a key point as input and the corresponding key point as output. This results in a smaller training data set of only 176 samples. In terms of the structure of input and output, this corresponds to a summary of similar statements to a key point in the context of a political debate on Twitter. Wolhandler et al. (2022) have found out that fine-tuning based on a single concatenated text rather than multiple source documents per training sample achieves a better performance. Details of the fine-tuning can be found in the Github repository.

### 2.3 Twitter data preparation

#### 2.3.1 Data collection

**Figure 3:**
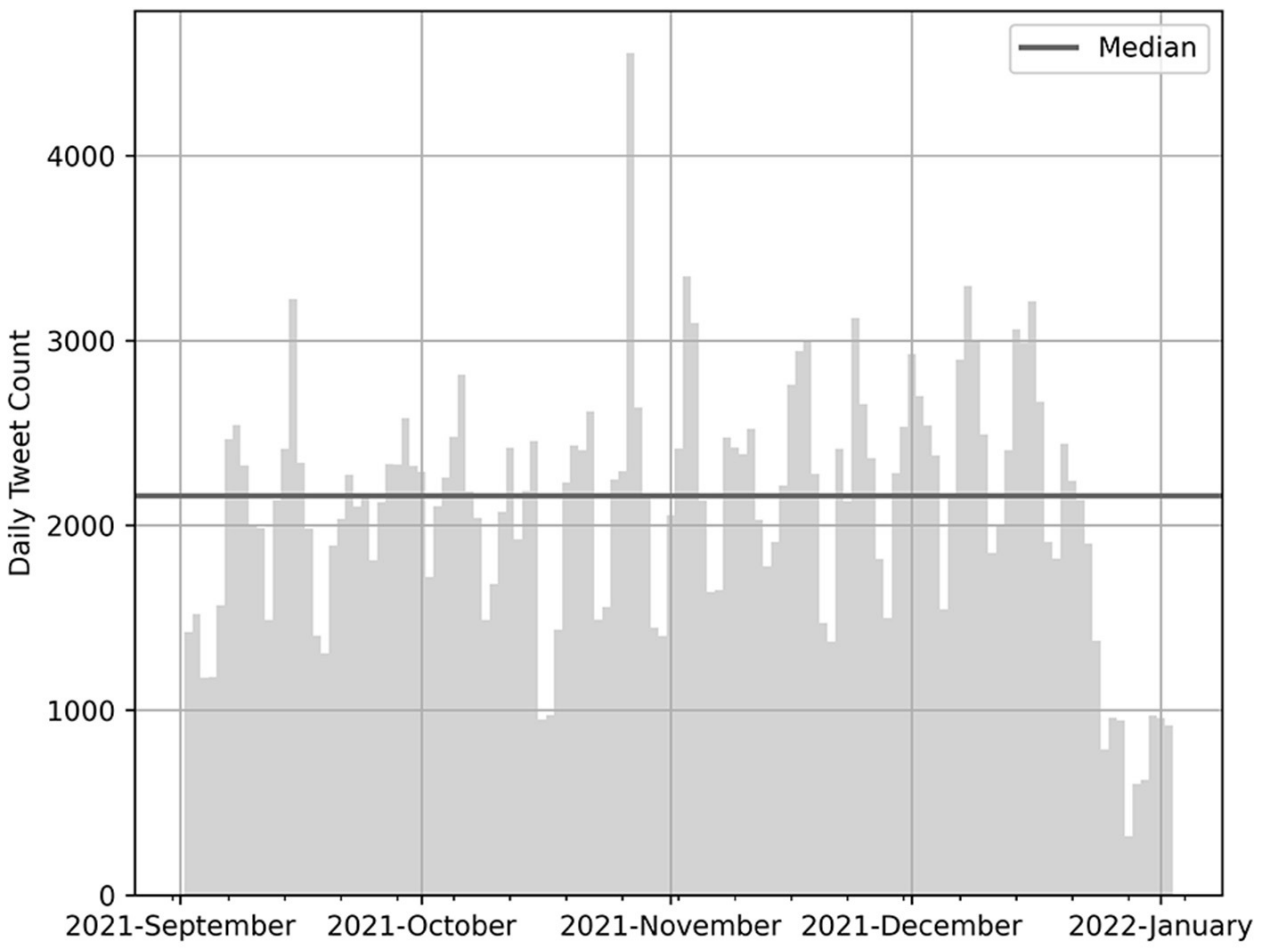
Number of daily tweets of British MPs retrieved via Twitter API between 2 September 2021 and 2 January 2022.

One of the aims of our research is to apply key point generation to the previously unaddressed domain of social media data. In order to collect a text corpus consisting of controversial topics and characteristics similar to the *ArgKP*_2021 data set, we collected 258, 184 statements of British parliamentarians active on Twitter between 3 September 2021 and 3 January 2022 using the Twitter API.^1^ We took the account data of Members of Parliament (MPs) from a freely available source,^2^ which contains, in addition to the account names, other metadata of interest for the analysis, such as party affiliation and constituency. We performed the iterative collection of Twitter data at 12-h intervals on an AWS EC2 instance using the *tweepy* library (see Figure 3). In addition to the tweet ID, timestamp, tweet text, and username, we added information on the party affiliation and constituency of each MP from the above source to the raw data and stored it in a MySQL database. The code, the account data of the MPs, and the structure of the SQL table are available in the Github repository.

#### 2.3.2 Data pre-processing

In a first step of the pre-processing pipeline, our research only considers tweets written between 2 September 2021 and 2 January 2022. We filtered out Retweets and duplicates, converted the text to lower case, and removed URLs and mentions. Neither did we remove stop words, nor did we apply stemming or lemmatization. These pre-processing steps do not add value to further processing using word embeddings (Schofield et al., 2017; Camacho-Collados and Pilehvar, 2018; Hickman et al., 2022) but may result in a loss of information. We excluded parties represented by < 1,000 tweets. In a final step, this research uses a fine-tuned RoBERTa language model (see Section 2.2.1) to perform a binary classification into non-argumentative and argumentative tweets.

#### 2.3.3 Topic modeling

In a final step of Twitter data preparation, we used the BERTopic framework (Grootendorst, 2022) to segment the pre-selected and now pre-processed tweets into rough topics. Following the procedure of Grootendorst (2022), the tweets are transformed into word embeddings by using the SBERT transformer *all*_*mpnet*_*base*_*v*2 (Reimers and Gurevych, 2019). In the second step, a dimensional reduction is performed using UMAP (McInnes et al., 2018) to prepare the input for the subsequent HDBSCAN clustering (Campello et al., 2013, 2015). Besides the minimum cluster size, set to 2% of the data set size, we used the standard parameters of the BERTopic framework. The clustering results were generated using the scripts of the Github repository.

### 2.4 Key point generation

#### 2.4.1 Topic selection

In order to perform key point generation on the Twitter data set created in chapter 2.3, we considered only one of the previously generated topics per run. According to Bar-Haim et al. (2020a), the aim of a key point analysis is to compare the arguments of two polarizing parties in the form of a list condensed into key points. Contrary to previous research (Bar-Haim et al., 2020a,b, 2021; Friedman et al., 2021), we did not determine the polarity of the two opposing parties by stance or sentiment on a topic but by party affiliation. The aim of the topic selection was to create a subset consisting of the statements of a political party on one of the latent topics identified in the course of topic modeling. Therefore, for all the topics listed in the Table 2, key points can be generated for the political parties Conservative, Labor, Liberal Democrat, and Scottish National Party.

#### 2.4.2 Sub-topic modeling

##### 2.4.2.1 Objective

Our research followed the study of Reimers et al. (2019), who semantically segment the data space based on statement similarity. To achieve this segmentation, we used a procedure based on the BERTopic (Grootendorst, 2022) framework. This approach aims at identifying similar statements in an unsupervised manner, in order to summarize them in the subsequent step of key point summarization using an abstractive method. Furthermore, the BERTopic procedure makes it possible to quantify the statements by cluster affiliation. Although this does not meet the requirements of the original definition of key point matching (Bar-Haim et al., 2020a) in terms of granularity, it does provide an approximate overview of the distribution of semantically similar statements in a text collection. To ensure a certain stability of the topic modeling with respect to the adaptation to unknown data and data structures, our research complemented topic modeling with hyperparameter optimization. The code for the corresponding AzureML HyperDrive implementation is available in the Github repository.

##### 2.4.2.2 Word Embeddings

To determine the similarity of tweets for further processing, they are transformed into corresponding 768 dimensional vector representations via SBERT transformer *all*_*mpnet*_*base*_*v*2 (Reimers and Gurevych, 2019), a procedure that corresponds to (Grootendorst, 2022). In a recent benchmark study (Muennighoff et al., 2023), the clustering and semantic textual similarity performance of this small-scale transformer, pre-trained mainly on user-generated data, was demonstrated. Following Reimers et al. (2016), we used the cosine distance *d*_*C*_ (see Equation 1) as a distance measure for pairwise determination of the distance between two vectors $\vec{a}$ and $\vec{b}$.


(1) $$\begin{array}{l} d_{C}\left(\vec{a},\vec{b}\right)=1-\frac{\vec{a}\cdot \vec{b}}{\left||\vec{a}||\cdot ||\vec{b}|\right|} \end{array}$$

In the equation above, the symbols are defined as follows:

- $d_{C}\left(\vec{a},\vec{b}\right)$ is the cosine distance between vectors $\vec{a}$ and $\vec{b}$,
- $\vec{a}$ and $\vec{b}$ are vector representations of tweets,
- $\vec{a}\cdot \vec{b}$ denotes the dot product of vectors $\vec{a}$ and $\vec{b}$,
- $\left||\vec{a}|\right|$ and $\left||\vec{b}|\right|$ represent the Euclidean norm (magnitude) of vectors $\vec{a}$ and $\vec{b}$, respectively.

##### 2.4.2.3 Dimension reduction

The effectiveness of the UMAP dimension reduction method on the cluster quality of subsequent HDBSCAN clustering has recently been described (Allaoui et al., 2020). According to McInnes et al., the reduction of high-dimensional data to low-dimensional representations also had a positive effect on the computation time of the HDBSCAN clustering procedure (McInnes and Healy, 2017). When using UMAP, the adjustment of hyperparameters has a significant influence on the final low-dimensional mapping of the data space. The most important parameters are the definition of the distance metric, the number of neighbors considered in the original data space, the number of desired target dimensions, and the height of the distance between the final low-dimensional representatives. To determine the distance of the data points in the original data space, we used the cosine distance (see Equation 1) because it is suitable for comparing high-dimensional data. UMAP allows greater control over the distance between the generated low-dimensional representatives than alternative dimension reduction methods such as t-SNE (van der Maaten and Hinton, 2008). We set the distance to 0, so that similar tweets had the highest possible density concentration in the target data space and are thus clearly separated for the subsequent clustering as described by Allaoui et al. (2020). In order to map relationships between data points in the original UMAP data space in a likelihood graph, the number of neighbors to be considered for the similarity calculation must also be specified. Since both the number of neighbors considered to generate the high-dimensional image and the desired number of target dimensions depend strongly on the nature of the data set being analyzed, we optimized these two parameters during hyperparameter tuning (see Section 2.4.2.5).

##### 2.4.2.4 Clustering

Each generated key point should semantically cover as many homogeneous statements as possible to reflect a single aspect of a debate (Bar-Haim et al., 2021). Therefore, we used the density-based clustering method HDBSCAN (Campello et al., 2013) to identify density centers of semantically similar statements without prior knowledge of the number of relevant density centers. Data points within regions of lower density are automatically labeled as noise (Campello et al., 2013). On the one hand, this has the advantage that only similar statements with a sufficient density concentration are considered for further key point summarization. On the other hand, it reduces the influence of the data noise often described in Twitter data (Barbosa and Feng, 2010; Derczynski et al., 2013) by excluding tweets that cannot be clearly classified.

The HDBSCAN clustering is mainly influenced by four parameter settings. A Euclidean distance metric (see Equation 2) is used to measure the distance between data points in the UMAP dimension-reduced data space.


(2) $$\begin{array}{l} d_{E}\left(\vec{a},\vec{b}\right)=\sqrt{\overset{n}{\underset{i=1}{\sum }}{\left(b_{i}-a_{i}\right)}^{2}} \end{array}$$

In the equation above, the symbols are defined as follows:

- $d_{E}\left(\vec{a},\vec{b}\right)$ represents the Euclidean distance between the points $\vec{a}$ and $\vec{b}$,
- $\vec{a}$ and $\vec{b}$ denote the position vectors of the two points in the n-dimensional UMAP reduced space, with components *a*_*i*_ and *b*_*i*_, respectively,
- The summation $\overset{n}{\underset{i=1}{\sum }}{\left(b_{i}-a_{i}\right)}^{2}$ calculates the square of the differences between the corresponding components of points $\vec{a}$ and $\vec{b}$,
- *n* is the dimensionality of the space after dimension reduction via UMAP.

In order to understand the remaining parameter settings, the functionality of HDBSCAN is explained in more detail. First, the mutual reachability distance *d*_mr_ (Campello et al., 2013) is determined to assign data points to regions of low or high density. *d*_mr_ of the two data points *a* and *b*, the core distance c_*k*_(*a*) or c_*k*_(*b*), and the parameter *k* must be determined. The parameter *k* represents the number of neighboring data points that have to be reached starting from a point *x* before its core distance c_*k*_(*x*) can be determined. The core distance c_*k*_(*x*) corresponds to the radius which in turn corresponds to the Euclidean distance from *x* to the *k*-nearest neighbor. To provide a basis for classifying data points into low or high density, the mutual reachability distance *d*_mr−*k*_ is calculated dependent on the parameter *k*. The determination of *d*_mr−*k*_(*a, b*) (see Equation 3) of the two data points *a* and *b* is done by selecting the maximum value of the core distance c_*k*_(*a*), the core distance c_*k*_(*b*), and the Euclidean distance *d*_*E*_(*a, b*).


(3) $$\begin{array}{l} d_{\text{mr}-k}\left(a,b\right)=\max\left\{{\text{c}}_{k}\left(a\right),{\text{c}}_{k}\left(b\right),d_{E}\left(a,b\right)\right\} \end{array}$$

In the equation above, the symbols are defined as follows:

- *d*_mr−*k*_(*a, b*) is the mutual reachability distance between data points *a* and *b*,
- c_*k*_(*x*) denotes the core distance of a point *x*, defined as the Euclidean distance from *x* to its *k*-nearest neighbor,
- *k* is the parameter indicating the minimum number of neighbors required to consider a point's core distance,
- *d*_*E*_(*a, b*) represents the Euclidean distance between points *a* and *b*.

Prim's algorithm (Prim, 1957) is then used to generate a weighted graph in the form of a minimum spanning tree, which defines data points as vertices and weights edges between data points according to *d*_mr−*k*_. By sorting the edges in ascending order based on their weight, a cluster hierarchy is created. The next step is to compress the fine-grained hierarchy using the *min*_*pts*_ parameter to determine the minimum cluster size. Finally, as described in the research study by Campello et al. (2013), the resulting clusters are extracted based on their cluster stability. In our research, we used the leaf method to extract all leaf nodes to subdivide the data space as finely as possible.

We set the distance metric and the clustering method as described. We set the parameter *min*_*pts*_ to one fiftieth of the size of the data set, so that only clusters representing a significant proportion of a debate are formed. In our research, only the parameter *k*, which determines the *k*-nearest neighbors to compute the core distance c_*k*_(*x*), was optimized by hyperparameter tuning.

##### 2.4.2.5 Hyperparameter tuning

Our research used hyperparameter optimization to stabilize the automatic detection of all relevant density centers of similar tweets through topic modeling. The definition of an optimal clustering strongly depends on the examined data characteristics and the actual use case (Hennig, 2015). Accordingly, the selection of parameters to be optimized, the sampling method, and the evaluation metric for determining the model quality must be individually adapted to the use case.

**Figure 4:**
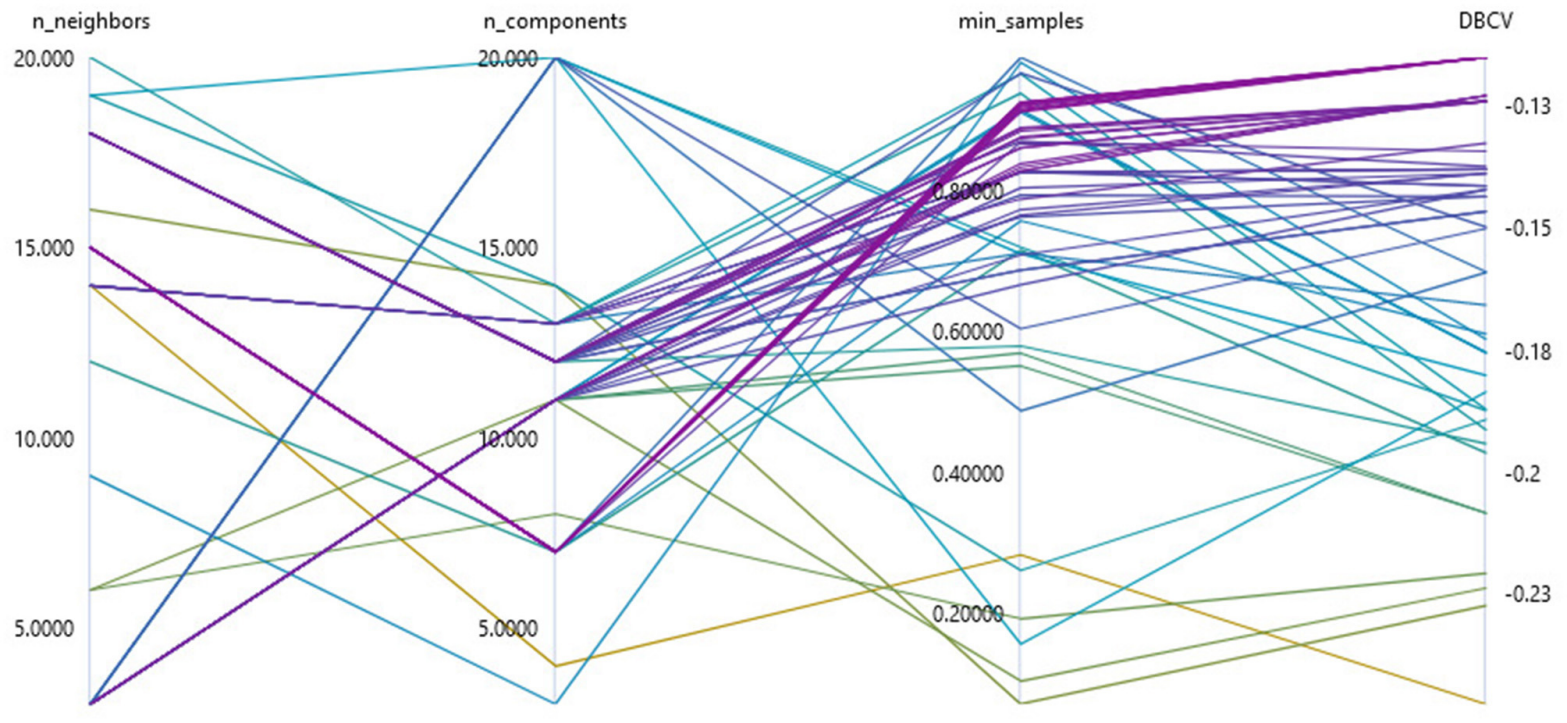
Visualization of the hyperparameter optimization of the sub-topic modeling using a Bayesian sampling for the parameterization of UMAP and HDBSCAN.

In our research, a total of three parameters were optimized to apply topic modeling to data with unknown structures (see Figure 4). For the UMAP method, we determined the optimal number of neighbors considered to create the high-dimensional image in the interval of [3, 20] and the optimal number of target dimensions in the interval of [3, 20]. To optimize the hyperparameters without prior knowledge of the structure of the data set under consideration, we used parameters that represent a multiplier of the data set size or the minimum cluster size. We determined the parameter *k* of the HDBSCAN clustering by the product of the minimum cluster size *min*_*pts*_ and the hyperparameter to be optimized *k*_*f*_ in the interval of [0.05, 1]. We chose this procedure because the parameter *k* is limited to the minimum cluster size. We set the value of the minimum cluster size as described above, to one fiftieth of the data set size.

In our research, a sampling method based on a Bayesian optimization with a Gaussian process (Snoek et al., 2012) had been chosen so that the hyperparameters were optimized with regard to the required hardware resources and convergence. Specifically, we applied the HyperDrive implementation of Bayesian sampling within an AzureML cloud environment (Ranjit et al., 2019) using a *Standard*_*NC*6*S*_*v*2 compute cluster with four compute nodes. The selection of parameters from a previously defined hyperparameter space is carried out by considering all evaluation results of previous procedures. This allows a probabilistic determination of which parameter configuration has the highest probability of optimizing model performance. Bayesian sampling achieves a significantly optimized parameter configuration with only a few samples (Wu et al., 2019).

In the course of Bayesian optimization, the quality of the model is determined by an evaluation metric. Density-based clustering algorithms such as HDBSCAN, with relatively high noise, pose special requirements for a target metric (Palacio-Niño and Berzal, 2019). Evaluation metrics for unsupervised clustering such as the silhouette coefficient (Rousseeuw, 1987), the Calinski-Harabasz index (Caliński and Harabasz, 1974), or the Davies-Bouldin Index (Davies and Bouldin, 1979) determine the cluster quality from the ratio of intra-cluster dispersion and inter-cluster separation. However, dispersion and separation are determined by a distance metric and are therefore not suitable for evaluating density-based methods (Palacio-Niño and Berzal, 2019). They also do not handle noise adequately (Moulavi et al., 2014). Given the characteristics of density-based methods in the context of hyperparameter optimization, we used density-based clustering validation (DBCV) as the optimization metric (Moulavi et al., 2014). The *DBCV* index in the interval of [−1, 1] also takes into account the influence of noise by considering all data points in the evaluation of the global cluster validity (Moulavi et al., 2014).

#### 2.4.3 Key point summarization

##### 2.4.3.1 Key Point Generation as abstractive summarization

Our research proposes a key point summarization once the data space has been segmented into semantic density centers. To generate a key point, we used abstractive methods to summarize the semantic content of identified sub-topics in a concise sentence. Previously, key points have often been generated using extractive summarization methods (Bar-Haim et al., 2020b, 2021), selecting highly representative and high-quality statements from the text collection itself. However, individual tweets rarely represent the entire content spectrum of their cluster, a fact partly explained by the non-convex shape of density centers generated by sub-topic modeling. They also rarely meet formal requirements such as word length and structure. In contrast, abstractive summarization methods are able to generate a coherent summary without redundancies but with the appropriate information density and shape (Gupta and Gupta, 2019). To implement such an abstractive approach, we fine-tuned a PEGASUS (Zhang et al., 2020) language model, as described in Chapter 2.2.2.

##### 2.4.3.2 Parameter setting

To generate concise key points, we set the maximum word length of the key points to 25. Our research determined the optimal minimum word length during hyperparameter tuning in an interval of [8, 20]. This ensured that key points have a sufficient number of words to represent the content of their cluster. Furthermore, we optimized the hyperparameter *p* in the interval [0.2, 0.95], which represents a probability threshold for selecting potential candidates for generating the next word of a sequence during the top-p sampling (Holtzman et al., 2019) procedure. In each step of sequence generation, the smallest possible pool of potential word candidates is selected whose cumulative probability for a given vocabulary *V* exceeds the threshold value *p*. According to Holtzman et al. (2019), the pool of potential word candidates *V*_*p*_ for each generated word *x* in a sequence of length *i* is determined as follows:


(4) $$\begin{array}{l} \underset{x\in V_{p}}{\sum }P\left(x|x_{1:i-1}\right)\geq p \end{array}$$

In the equation above, the symbols are defined as follows:

- *p* denotes the probability threshold for selecting potential word candidates during the top-p sampling process,
- *V* represents the entire vocabulary from which word candidates are chosen,
- *V*_*p*_ is the subset of *V* consisting of word candidates whose cumulative probability meets or exceeds the threshold *p*,
- *x* refers to a potential word candidate within the sequence,
- *x*_1:*i*−1_ denotes the sequence of words generated up to the current step *i*−1,
- *P*(*x*|*x*_1:*i*−1_) is the conditional probability of the candidate word *x* given the sequence of previously generated words *x*_1:*i*−1_.

Furthermore, top-p sampling is combined with top-k sampling (Fan et al., 2018). The parameter *k*, which is optimized in the interval [10, 100], indicates the maximum number of potential word candidates that will be considered for each step of sequence generation. This additional parameter prevents words with a very low probability from being considered as word candidates in the course of top-p sampling.

Since the maximum input sequence length of the language model is limited to 512 tokens, we filtered the most representative statements for each cluster. Therefore, we calculated the average cosine distance of each tweet to all other tweets in its cluster. In a final step, we concatenated the statements with the lowest average cosine distance up to a token length of 512.

##### 2.4.3.3 Hyperparameter tuning

**Figure 5:**
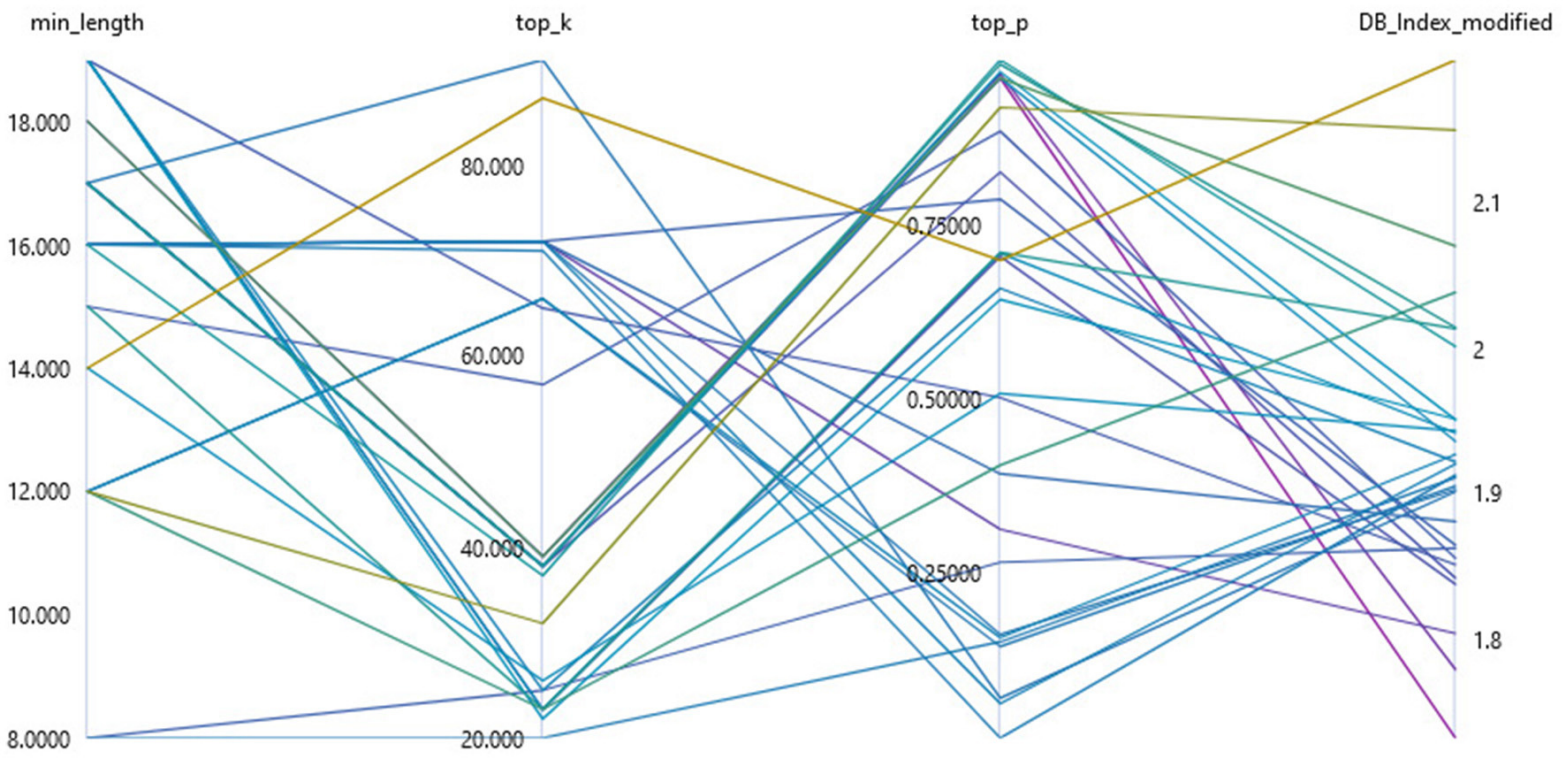
Hyperparameter optimization of key point summarization using sampling parameters for the PEGASUS language model using the modified Davies-Bouldin Index as an evaluation metric.

Automated evaluation of key point summarization is not trivial. Previous research has taken different approaches to evaluating the results of key point or opinion summarization: An automatic evaluation via the ROUGE value (Bražinskas et al., 2020a,b), a human evaluation (Suhara et al., 2020; Friedman et al., 2021) or by using a labeled ground truth data set and the obtained precision and coverage (Bar-Haim et al., 2020b, 2021). Since human evaluation in the course of hyperparameter tuning was excluded and since the aim of our research is an unsupervised application of key point generation to unknown data, two of the above options were already omitted. In order to perform an automated evaluation that does not assess the quality of the summaries at the word level, as the ROUGE metric (Lin, 2004) does, but also at the semantic similarity of the key points generated, this research performed an automated evaluation on the basis of word embeddings. As for the topic modeling, we used the SBERT Transformer *all*_*mpnet*_*base*_*v*2 (Reimers and Gurevych, 2019) and the cosine distance to determine the semantic similarity. We evaluated automated key point summarization (exemplarily shown in Figure 5) using a modified version of the Davies-Bouldin Index (Davies and Bouldin, 1979) proposed in this research. This index, originally designed to assess cluster quality, is calculated from the ratio of intra-cluster dispersion and inter-cluster separation. We calculated the intra-cluster dispersion *S*_*i*_ (see Equation 5) from the average cosine distance *d*_*C*_ of all data points *T*_*i*_ of a cluster *i* to the cluster centroid *A*_*i*_. Since a key point should semantically cover all data points in its cluster, we considered it to be the centroid of its cluster. A low cluster dispersion therefore suggests a key point that represents the semantics of its own cluster well.


(5) $$\begin{array}{l} S_{i}=\frac{1}{T_{i}}\overset{T_{i}}{\underset{j=1}{\sum }}d_{C}\left(X_{j},A_{i}\right) \end{array}$$

In the equation above, the symbols are defined as follows:

- *S*_*i*_ represents the intra-cluster dispersion for cluster *i*, indicating the average distance of all data points within the cluster to the cluster centroid,
- *T*_*i*_ denotes the total number of data points within cluster *i*,
- *d*_*C*_(*X*_*j*_, *A*_*i*_) is the cosine distance between a data point *X*_*j*_ and the cluster centroid *A*_*i*_,
- *X*_*j*_ refers to the *j*-th data point within cluster *i*,
- *A*_*i*_ is considered the centroid of cluster *i*, representing the key point that semantically covers all data points in its cluster.

We modified the calculation of cluster separation described by Davies and Bouldin (1979) and calculated the cluster separation *M* for each combination of generated key points (see Equation 6). We derived the cluster separation *M*_*ij*_ of the two clusters *i* and *j* by calculating the cosine distance *d*_*C*_ of the two cluster key points *A*_*i*_ and *A*_*j*_. This is in line with the aim of generating a list of key points, which should not be redundant and should each represent only one aspect of a debate (Bar-Haim et al., 2021).


(6) $$\begin{array}{l} M_{ij}=d_{C}\left(A_{i},A_{j}\right) \end{array}$$

In the equation above, the symbols are defined as follows:

- *M*_*ij*_ denotes the separation between clusters *i* and *j*, intended to measure the dissimilarity between the clusters' key points,
- *d*_*C*_(*A*_*i*_, *A*_*j*_) represents the cosine distance between the key points *A*_*i*_ and *A*_*j*_ of clusters *i* and *j*, respectively,
- *A*_*i*_ and *A*_*j*_ are the centroids of clusters *i* and *j*, effectively serving as the key points that summarize the main concepts of their respective clusters.

Davies and Bouldin (1979) determine the relation *R* between cluster dispersion and cluster separation for each cluster combination (see Equation 7). The relation *R*_*ij*_ of the two clusters *i* and *j* is defined by the ratio of the sum of the cluster dispersion *S*_*i*_ and *S*_*j*_ to the corresponding cluster separation *M*_*ij*_.


(7) $$\begin{array}{l} R_{ij}=\frac{S_{i}+S_{j}}{M_{ij}} \end{array}$$

In the equation above, the symbols are defined as follows:

- *R*_*ij*_ quantifies the relation between the dispersion within clusters *i* and *j* and the separation between them, serving as a measure of clustering efficiency,
- *S*_*i*_ and *S*_*j*_ represent the intra-cluster dispersion for clusters *i* and *j*, respectively, indicating the average distance of data points within each cluster to their centroid,
- *M*_*ij*_ denotes the separation between clusters *i* and *j*, calculated as the cosine distance between their respective centroids (key points).

According to Davies and Bouldin (1979), the worst relation *R*_*i*_ is determined for each cluster *i* by taking the maximum value of all its cluster relations. Finally, the Davies-Bouldin Index $\bar{R}$ is calculated by taking the average of the worst relations *R*_*i*_ of all the clusters (see Equation 8).


(8) $$\begin{array}{l} \bar{R}=\frac{1}{N}\overset{N}{\underset{i=1}{\sum }}R_{i} \end{array}$$

In the equation above, the symbols are defined as follows:

- $\bar{R}$ represents the Davies-Bouldin Index, which serves as a measure of the quality of clustering based on cluster dispersion and separation,
- *R*_*i*_ is the worst relation for cluster *i*, determined as the maximum relation among all cluster relations for that cluster,
- *N* denotes the total number of clusters in the clustering result.

### 2.5 Evaluating key point generation

To determine the quality of key point generation in an automated approach, our research considered the procedure as a multidocument summarization task. Quality of a multidocument summarization is assessed by comparing a generated summary with a reference summary of all documents. Since there is no generated summary available, we considered the concatenated list of all generated key points of a topic to be the generated summary. Similarly, we derived the reference summary from the concatenation of the key points of a topic, manually annotated by experts in the field.

In our research, the evaluation was based on the *ArgKP*_2021 data set, which had already been used as a training data set for abstractive summarization (see Section 2.2.2). For the evaluation, we only considered six topics of the data set that had not been used in the model training. Since the topics are additionally divided into arguments with a positive or negative stance toward the topic, we included a total of twelve subsets in the evaluation.

We used the word-based metric ROUGE (Lin, 2004) and the word-embedding-based metric BERTScore (Zhang T. et al., 2019) as evaluation criteria. We chose ROUGE because it has become the standard metric for evaluating summaries (Fabbri et al., 2021). The lexical similarity is calculated in the course of the evaluation on the basis of unigrams (ROUGE-1) and bigrams (ROUGE-2).

To evaluate beyond the lexical dimension, we complemented the assessment with BERTScore. This metric computes a cosine similarity between the corresponding word embeddings for each pairwise token of the generated and reference summaries. Thus, the metric is able to detect semantic overlap between the reference and the generated summary even when there is no lexical overlap (Zhang T. et al., 2019). We chose “microsoft/deberta-xlarge-mnli” (He et al., 2021) as the underlying model for generating word embeddings as it has the highest correlation with human evaluation in terms of predicted similarity (Zhang T. et al., 2019). The BERTScore is given in the interval of [0, 1], where a higher value represents a higher precision, recall, or f1 score.

Our research carried out the evaluation per topic and stance of the evaluation data set. Only statements that can be explicitly assigned to a topic were taken into account. In order to perform the evaluation on subsets with an average number of 90 statements, we had to adjust the minimum cluster size parameter from one fiftieth to one twentieth of the data set under consideration. The code for the corresponding AzureML HyperDrive implementation of the evaluation is available in the Github repository.

## 3 Results

### 3.1 Twitter data preparation

The first aim of our research was to prepare social media data for an exemplary application of key point generation to a political debate on Twitter. A total of 258, 184 tweets from British MPs were obtained via the Twitter API between the 2 September 2021 and 3 January 2022. During the pre-processing pipeline (see Table 1), the number of tweets was reduced to 34, 998. In particular, the two steps of filtering retweets and duplicates (257,149 → 120,419) and non-argumentative tweets (114,475 → 34,998) significantly reduced the data volume. To identify latent topics, we segmented the remaining data set into rough clusters using topic modeling (Grootendorst, 2022). This resulted in a total of nine topics with an average number of 2, 049 tweets and 16, 559 statements that were not assigned to any cluster (see Table 2).

**Table 1:** Overview of each pre-processing step as well as the absolute number and relative proportion of the remaining tweets in the raw data set.

**Pre-processing pipeline**	
**Processing step**	**Number tweets**
Raw data extraction via Twitter API	258,184 (100%)
Delimitation of the survey period	257,149 (99.6%)
Filter retweets and duplicates	120,419 (46.6%)
Filter URLs and mentions	118,862 (46%)
Filter parties with < 1,000 tweets	114,475 (44.3%)
Filter non-argumentative tweets	34,998 (13.6%)

**Table 2:** Topics of the political debate on Twitter identified within the data set, including associated number of tweets and topic label.

**Topic**	**Count**	**Topic label**
0	3,413	social_care_tax_universal
1	2,634	great_local_work
2	2,445	climate_cop_green_energy
3	2,379	uk_people_trade
4	2,286	women_violence_police
5	2,143	covid_vaccine_booster
6	1,588	rules_MPs_tory_corruption
7	849	rail_transport_north_services
8	702	remembrance_armed_service
−1	16,559	Not assigned to a topic

### 3.2 Sub-topic modeling

**Figure 6:**
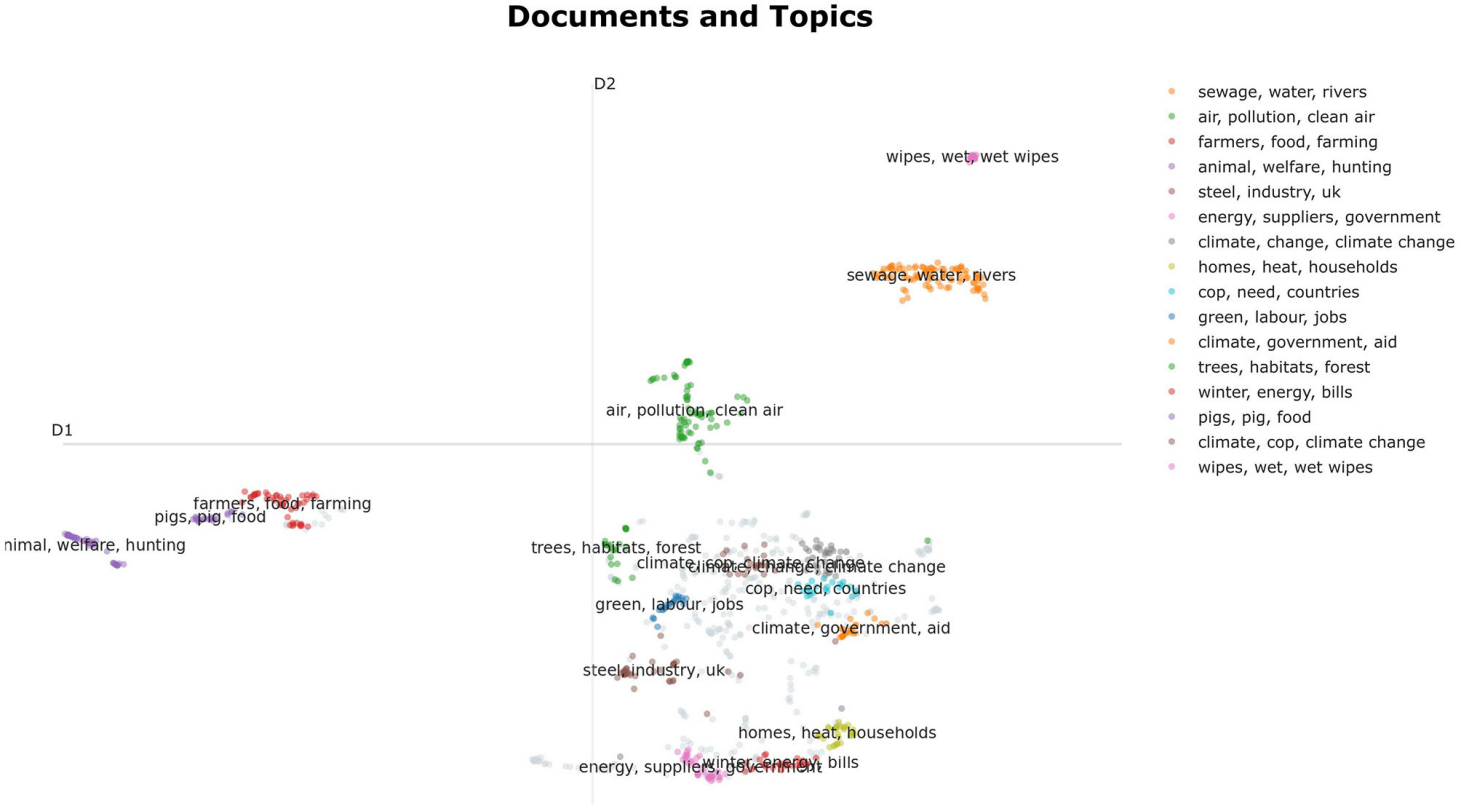
Visualization of a semantically segmented debate of Labor tweets on the topic of climate change using the hyperparameter-optimized topic modeling approach. For representation purposes, the data space was reduced to two dimensions using BERTopic (Grootendorst, 2022).

The next research objective was to develop a method to identify clusters of semantically similar tweets, as a basis for the subsequent key point summarization. For this purpose, we used a customized variant of BERTopic (Grootendorst, 2022), which we complemented by hyperparameter tuning. In the course of this procedure, we considered statements of one party on a specific topic. To perform topic modeling, we generated word embeddings of the statements using an SBERT transformer (Reimers and Gurevych, 2019). Then, we used UMAP (McInnes et al., 2018) to reduce the dimensions of the embeddings to prepare them for the subsequent HDBSCAN clustering (Campello et al., 2013) as suggested by Grootendorst (2022). We used the evaluation metric DBCV (Moulavi et al., 2014) to optimize relevant parameters of UMAP and HDBSCAN during hyperparameter tuning. We set parameters that depend on the size and structure of the data set relative to the size of the data set, so that the procedure could be automatically applied to unknown data without obvious limitations. As an example, the procedure of sub-topic modeling was applied to the Labor Party's statements on the topic “climate_cop_green_energy”. The result of the sub-topic modeling was visualized in a dimensionally reduced representation using the BERTopic framework (see Figure 6).

### 3.3 Key point generation

The main goal of key point generation is to create a list of key points that semantically cover a party's central statements on a given topic. Therefore, our research used the textual cluster contents of the sub-topic modeling results and concatenated them until the maximum input sequence length of the PEGASUS language model (Zhang et al., 2020) was reached. We prioritized statements based on their average cosine similarity to other statements in their cluster. We then summarized the concatenated statements using a PEGASUS language model that had been fine-tuned specifically for this use case. Our research evaluated KPG using a modified Davies-Bouldin Index (Davies and Bouldin, 1979), which calculates cluster dispersion and separation using the average distance to each key point rather than cluster centroids. We used this metric to optimize the minimum sequence length and the sampling parameters of the sequence generation in the course of hyperparameter tuning. Since each key point is supposed to represent the contents of its cluster, we considered the corresponding cluster size as an indicator of the key point's share in the overall debate.

To assess the quality of the key point generation, we performed an automated evaluation using the *ArgKP*_2021 data set. The approach taken in our research treats KPG as a summarization problem, where the concatenation of key points represents the summary 2.5. The quantitative results (see Table 3) show that the number of generated key points strongly deviates from the number of key points annotated by experts. Furthermore, the recall values for the evaluation metric BERTScore are on average higher than for the corresponding precision values. Finally, the table shows that the ROUGE metric has a high variance compared to the corresponding BERTScore value.

**Table 3:** Results of the evaluation of key point generation based on the (ArgKP_2021) data set using the metrics ROUGE-1, ROUGE-2, and BERTScore (Precision, Recall, f1).

**Topic**	**Stance**	**ROUGE**		**BERTScore**			**Number key points**	
		**1**	**2**	**P**	**R**	**f1**	**given**	**generated**
The USA is a good country to live in	Positive	31.02	6.49	0.57	0.66	0.62	7	8
	Negative	16.67	2.8	0.54	0.63	0.58	7	11
Social media platforms should be regulated by the government	Positive	39.52	13.33	0.57	0.6	0.65	5	9
	Negative	37.13	14.55	0.57	0.66	0.61	5	10
Routine child vaccinations should be mandatory	Positive	31.72	16.78	0.62	0.69	0.66	5	9
	Negative	23.07	7.78	0.5	0.63	0.56	4	9
The vow of celibacy should be abandoned	Positive	22.95	6.67	0.63	0.73	0.67	5	7
	Negative	41.07	12.73	0.64	0.69	0.66	6	5
Assisted suicide should be a criminal offense	Positive	31.28	6.7	0.59	0.64	0.61	4	10
	Negative	29.71	12.72	0.56	0.69	0.62	6	12
Homeschooling should be banned	Positive	17.71	4.21	0.49	0.61	0.54	4	11
	Negative	43.14	11.88	0.61	0.68	0.64	6	8
								
**Average:**		30.42	9.72	0.57	0.66	0.61	5.33	9.08

We analyzed an exemplary result of a KPG on the topic of celibacy (see Supplementary Table S1) qualitatively. Overall, the key points that are generated largely reflect the semantics of the annotated key points. However, the generated key points generally show an increased number of words, occasional semantic redundancies, and partially uncovered content of the reference key points. In addition, a high number of statements are not assigned and the quantification of the key points differs greatly from the information provided by the experts. In order to analyze the adaptability to the domain of political debate on Twitter, we applied KPG to Conservative and Labor statements on the topic of “climate_cop_green_energy”, which roughly covers all topics related to climate change (see Supplementary Tables S2, S3). The generated key points largely meet the requirements of Bar-Haim et al. (2021) in terms of validity, informativeness and aspect focus. In some cases, however, the key points are formulated generically (see key point IDs in Supplementary Tables S2, S3 Conservative: 2,9,12 Labor: 3,14), they do not contain complete sentences (Conservative: 8) or they do not provide all the essential information (Labor: 8,10). Furthermore, some key points contain non-informative fragments of sentences and are often formulated subjectively.

## 4 Discussion

### 4.1 Conclusion

In this research, we have successfully implemented an innovative unsupervised method for KPG, aligned with the key point analysis paradigm proposed by Bar-Haim et al. (2020a, 2021). Our approach, based on topic modeling and enhanced by hyperparameter tuning, not only segments the data space but also approximates the frequency of statements associated with each key point, filling a gap in previous KPG methodologies (Bar-Haim et al., 2021). Our decision to adopt an abstractive summarization approach, as opposed to traditional extractive methods (Bar-Haim et al., 2020b, 2021; Alshomary et al., 2021), proved advantageous. It allowed for a broader semantic representation within key points, capturing different aspects of a subtopic more effectively. This was facilitated by optimizing the minimum sequence length during hyperparameter tuning, ensuring that key points more accurately reflected the content of the associated statements. Furthermore, we incorporated the Davies-Bouldin Index, modified to suit our research, into the hyperparameter tuning process. This procedure both optimizes the generated key points to better represent the semantic content of their cluster and at the same time differentiates them from other key points in terms of their semantic content, as examplarily shown in Figure 5.

Our research introduces an evaluation method for KPG that uses both ROUGE, a word-based metric, and BERTScore, a word- embedding-based metric, by considering KPG as a multidocument summarization problem. This dual-metric approach allows for a more nuanced evaluation of the generated key points, capturing not only lexical similarity but also semantic coherence with the source documents. By framing KPG in the context of multidocument summarization, we acknowledge the complexity of distilling key information from multiple statements, and our evaluation method is designed to reflect both the precision of word choice and the depth of contextual understanding.

In conclusion, while our methodology was specifically designed to analyse data from the dynamic and often unstructured environment of social media, its theoretical foundation suggests potential applicability to a variety of contexts. The unsupervised principles underlying our approach, particularly in dealing with diverse and complex datasets through density-based clustering and subsequent hyperparameter tuning, may provide valuable insights for domains such as product reviews and customer surveys. However, it is important to note that the direct applicability of our methods to these domains remains to be fully explored and validated. The initial success in the social media context lays a promising foundation and encourages further exploration of the adaptability of our KPG techniques. This exploration not only addresses our initial research question but also invites future studies to rigorously test and expand the reach of our methodology and evaluation in various fields of key point analysis.

### 4.2 Limitations

#### 4.2.1 Theoretical limitations

Apart from the advantages described above, our research has some theoretical limitations that need to be addressed. The quantitative evaluation revealed an overgeneration of key points compared to expert references, often leading to semantic redundancy, as reflected in the low precision of the BERTScore (see Table 3). This problem was less pronounced in denser semantic spaces such as political debates with more examples. In addition, the qualitative analysis showed that the key points generated sometimes did not match the criteria of Bar-Haim et al. (2021) (see Section 3.3). This suggests that treating key points as complete representations of clustered statements may be an oversimplification, especially given the observed semantic redundancy and deviation from established criteria.

Our research indirectly attempts to quantify the statements associated with a key point by approximating the cluster size, but this method deviates from the actual values 3.3. This discrepancy is due to the complexity of key point matching, where key points may not align exclusively with a single cluster. In addition, the use of HDBSCAN clustering often labels many statements as noise, making it difficult to accurately determine the relative prominence of a key points in political debates.

Our study also acknowledges the limitations of the ROUGE metric in evaluating abstractive approaches, as reflected in the variance of ROUGE scores in our results (see Table 3) and confirmed by previous research as humans tend to formulate abstractive summaries that are mostly not equivalent in terms of word choice (Schluter, 2017). The fluctuations of the ROUGE value observed in Chapter 3.3 can be attributed to a property of ROUGE.

Another theoretical limitation arises from the inherent nature of our methodology, which includes the stochastic aspect of topic modeling and subsequent hyperparameter tuning. This process, when run repeatedly, can produce different results on the same dataset due to its optimization dynamics, adding another layer of complexity and uncertainty to our research approach.

#### 4.2.2 Practical limitations

Our research has identified critical practical limitations that impact the effectiveness and broader applicability of our approach. A primary concern is the evaluation's credibility, which is compromised by our reliance on the scarcity of high-quality, rich datasets for short, opinionated statements. This limitation not only restricts the conclusiveness and generalizability of our findings but also severely hampers the fine-tuning of our language models. Such fine-tuning is crucial for effective key point generation. Consequently, this directly affects our ability to robustly validate the effectiveness of our topic modeling and hyperparameter tuning efforts, potentially compromising the accuracy and robustness of our results.

Second, the applicability of the KPG approach is somewhat limited, focussing primarily on social media data and political debates within the *ArgKP*_2021 dataset. This specialization limits the broader applicability of our findings across different data types or domains.

Finally, the inherent limitations of the language models we use, in particular their constrained input sequence length, have an impact on our methodology. This constraint forces us to use only cluster representatives of a certain token length for abstractive summarization, which limits the quality of summarization in terms of coverage.

These practical limitations highlight the need for continuous refinement and development of our approach and methods.

### 4.3 Future work

Acknowledging these practical limitations not only informs the current state of our research but also provides a clear path for our future research in the field of KPG. A key part of this endeavor will be an extensive comparative analysis using different datasets such as *ArgKP*_2021 (Bar-Haim et al., 2021), Opinosis (Ganesan et al., 2010), and *SPACE* (Angelidis et al., 2021). This study will not only benchmark our proposed KPG method against existing extractive methods (Bar-Haim et al., 2020b, 2021) but also explore its scalability and adaptability across different domains, data sizes, and types.

In parallel, we plan to enhance our evaluation techniques. While automated metrics such as BERTScore (Zhang T. et al., 2019) have been valuable, incorporating comprehensive human scoring will provide deeper qualitative insights. This combination of automated and human scoring will ensure a more holistic assessment of the key points generated.

Addressing the challenge of semantic redundancy in KPG is also critical. We aim to develop advanced algorithms and refine techniques within frameworks such as BERTopic (Grootendorst, 2022) to minimize redundancy. This effort will also include exploring post-processing strategies to enforce semantic distinctiveness, ensuring the uniqueness and relevance of each key point. We also intend to conduct a detailed evaluation using crowd annotators, inspired by the methodology of Friedman et al. (2021). This approach will help us identify specific error patterns and understand user perspectives on the relevance and quality of key points.

The exploration of advanced language models is another key aspect of our future work. We will be experimenting with models such as BART (Lewis et al., 2020), T5 (Raffel et al., 2020), BLOOM (Scao et al., 2022), and their long form variants such as BigBird PEGASUS (Zaheer et al., 2020) and LongT5 (Guo et al., 2022). Our focus will be on evaluating how these models, with their enhanced input sequence capabilities, can improve the depth and quality of KPG.

An important consideration in our research will be the evaluation of computational efficiency, especially when using advanced sentence similarity methods such as BERTScore (Zhang T. et al., 2019) and MoverScore (Zhao et al., 2019). We aim to balance the trade-off between performance improvements and resource requirements, taking into account the practical implications for large-scale applications.

There will also be a focus on methodological innovation, in particular on optimizing the clustering process and experimenting with new hyperparameter tuning approaches. Inspired by recent advances described in Bacanin et al. (2022), we will explore the use of meta heuristic techniques for hyperparameter tuning. These techniques, known for efficiently navigating complex parameter spaces, show promise for improving model performance. This approach is especially pertinent for tasks like abstractive summarization, where precise control over multiple parameters is crucial.

Finally, we plan to expand the scope of our research to include multilingual and cross-cultural contexts. This extension will involve adapting the KPG methodology to different languages and cultural settings, thereby broadening its applicability and relevance.

## Data availability statement

The raw data supporting the conclusions of this article will be made available by the authors, without undue reservation.

## Ethics statement

Ethical approval was not required for the study involving human data in accordance with the local legislation and institutional requirements. The social media data was accessed and analyzed using the Twitter API.

## Author contributions

JS: Writing—review & editing, Supervision, Conceptualization, and Project administration. All authors listed have made a substantial, direct, and intellectual contribution to the work and approved it for publication.

## Footnotes

^1^ https://developer.twitter.com/en

^2^ https://www.politics-social.com/
