# Supplementary material for: Key point generation as an instrument for generating core statements of a political debate on Twitter
Source: Front Artif Intell. 2024 Mar 20;7:1200949. doi: 10.3389/frai.2024.1200949 (PMC10993730; doi:10.3389/frai.2024.1200949)
Supplement: Supplementary file 1 [file Data_Sheet_1.PDF]

Supplementary Material

Stance	ID	Key Point	Size	%
**Evaluation results of topic ”The vow of celibacy should be abandoned”**				
**Generated Key Points**				
positive	0	The vow of celibacy should be abandoned because it is outdated/antiquated	12	11.8
	1	The vow of celibacy is preventing men from joining the priesthood/religious vocations	11	10.1
	2	The vow of celibacy is outdated and irrelevant to today’s sexual revolution	8	7.8
	3	The vow of celibacy harms the church/the person who is being forced to abstain	6	5.9
	4	Celibacy harms the church/sexualizes those who are in positions of sexual responsibility	6	5.9
	5	The vow of celibacy is an unnecessary restriction on sexual desire/risk-taking	6	5.9
	6	Celibacy is a major cause of sexual abuse in the catholic church/not just pedophilia	5	4.9
		**Statements not assigned:**	48	47.1
negative	0	Celibacy should not be abandoned as it is still important for some religious people	22	27.1
	1	Celibacy allows a priest to focus on his job and not worry about other issues	13	16
	2	The vow of celibacy is essential to become a priest/supreme cleric	8	9.8
	3	Celibacy is beneficial for the person making a vow - it increases self awareness	7	8.6
	4	A vow should not be changed because it is based on a religion’s beliefs	6	7.4
		**Statements not assigned:**	25	30.9
**Key Points annotated by experts**				
positive	0	Celibacy is outdated	46	45.1
	1	Celibacy is unhealthy/unnatural	20	19.6
	2	Celibacy leads to wrong sexual behaviour	19	18.6
	3	Celibacy is too pressuring/diffcult	10	9.8
	4	Celibacy reduces the pool of people wanting to become priests	8	8.8
negative	0	Celibacy allows a priest to focus on God	28	34.6
	1	Celibacy is a strong religious belief	18	22.2
	2	Religious experiences and traditions should be maintained	15	18.5
	3	Priests choose to be celibates	11	13.6
	4	Celibacy makes the priests to be perceived as holy	5	6.2
	5	Removing celibacy could lead to sexually reckless acts	4	4.9

Table S1. Qualitative evaluation result of the Key Point Generation on the basis of statements with a positive and negative stance towards the topic ”The vow of celibacy should be abandoned” of the data set $ArgKP\_2021$.

Party	ID	Key Point	Size	%
Conservative	0	The uk needs more energy production to avoid future shortages/high prices we should increase domestic output	85	7.9
	1	Today is backbritishfarmingday i'm proud to support our farmers	78	7.3
	2	As the uk prepares to meet its climate change commitments, here's a round-up of comments from key figures	76	7.1
	3	Pet theft is a terrible crime which affects the lives of pets and those who care for them	69	6.4
	4	The government has taken important steps to tackle sewage pollution in our waterways but more work is needed	69	6.4
	5	Nuclear power is a key part of our future energy mix good news on small modular reactors	62	5.7
	6	A selection of quotes from key figures at the flood actionweek conference in surrey	36	3.4
	7	As part of cop week i have been visiting schools and speaking to students about climate change	35	3.3
	8	As storm arwen continues to cause disruption in northumberland my team and i are closely monitoring the progress of	34	3.2
	9	A busy week in london for the uk maritime sector, here's a round-up of my highlights:	33	3.1
	10	Climate change is one of the most urgent problems we face and must urgently act to tackle it	31	2.9
	11	A net zero economy is a win-win for businesses, consumers and the environment	29	2.7
	12	A look at some of the key environmental initiatives we have been working on this week:	26	2.4
	13	Water pollution is not a cost-effective measure i people are misrepresenting votes in parliament as usual	25	2.3
	14	In practical real life terms the state of our drains is such that emergency decisions sometimes have to be made eg between houses	25	2.3
	15	The environment bill is already part of the government's armoury to tackle storm discharges	22	2.1
		**Outliers:**	340	31.6
		**Total Tweets:**	1075	

Table S2. An example of KPG's findings applied to the political debate on Twitter to provide a qualitative assessment of statements on climate change given by members of the Conservative party.

Party	ID	Key Point	Size	%
Labour	0	Raw sewage is being pumped into our rivers and seas - here are some of the views from MPs	97	10.4
	1	Air pollution kills more than a million people every year in the UK and needs to be tackled	75	8
	2	Farmers are under threat from cheap imports and the government must do more to protect their interests	46	4.9
	3	Here are some of the animal welfare issues that have been discussed by MPs this week:	40	4.3
	4	The government must take urgent action to protect the steel industry and create a green industrial strategy	37	4
	5	The collapse of energy companies shows that the government is not prepared to take responsibility for its failures	37	4
	6	The climate crisis is upon us and we have a choice to make/we can confront it	32	3.4
	7	The government's failure to deliver on the green homes grant shows a complete lack of commitment	27	2.9
	8	The summit is a critical moment for the world's leaders to deliver on their commitments	24	2.6
	9	A green new deal is needed to create good jobs and protect the environment here are some key points from Labour's	22	2.4
	10	Climate justice is not a priority for Boris the government has failed to take action	22	2.4
	11	The agreement is a step in the right direction but we need to see more concrete action	22	2.4
	12	The government must act now to protect vulnerable families from the impact of rising energy bills this winter	22	2.3
	13	The crisis in the pig sector is not just a result of incompetent government and Brexit	20	2.1
	14	A look at some of the highlights from our climate change events in London this week:	20	2.1
	15	Plastic in wet wipes harms the environment I support putneyfleurs bill to banplasticinwetwipe	19	2
		**Outliers:**	372	39.8
		**Total Tweets:**	934	

Table S3. An example of KPG's findings applied to the political debate on Twitter to provide a qualitative assessment of statements on climate change given by members of the Labour party.
